# Pharmacogenetic Foundations of Therapeutic Efficacy and Adverse Events of Statins

**DOI:** 10.3390/ijms18010104

**Published:** 2017-01-06

**Authors:** Elena Arrigoni, Marzia Del Re, Leonardo Fidilio, Stefano Fogli, Romano Danesi, Antonello Di Paolo

**Affiliations:** 1 Clinical Pharmacology and Pharmacogenetic Unit, Department of Clinical and Experimental Medicine, University of Pisa, Via Roma 55, 56126 Pisa, Italy; elena.arrigoni@hotmail.com (E.A.); marzia.delre@ao-pisa.toscana.it (M.D.R.); leofidilio@gmail.com (L.F.); romano.danesi@unipi.it (R.D.); 2 Department of Pharmacy, University of Pisa, Via Bonanno Pisano 6, 56126 Pisa, Italy; stefano.fogli@farm.unipi.it

**Keywords:** statins, pharmacogenetics, epigenetics, drug transporters, toxicity

## Abstract

Background: In the era of precision medicine, more attention is paid to the search for predictive markers of treatment efficacy and tolerability. Statins are one of the classes of drugs that could benefit from this approach because of their wide use and their incidence of adverse events. Methods: Literature from PubMed databases and bibliography from retrieved publications have been analyzed according to terms such as statins, pharmacogenetics, epigenetics, toxicity and drug–drug interaction, among others. The search was performed until 1 October 2016 for articles published in English language. Results: Several technical and methodological approaches have been adopted, including candidate gene and next generation sequencing (NGS) analyses, the latter being more robust and reliable. Among genes identified as possible predictive factors associated with statins toxicity, cytochrome P450 isoforms, transmembrane transporters and mitochondrial enzymes are the best characterized. Finally, the solute carrier organic anion transporter family member 1B1 (*SLCO1B1*) transporter seems to be the best target for future studies. Moreover, drug–drug interactions need to be considered for the best approach to personalized treatment. Conclusions: Pharmacogenetics of statins includes several possible genes and their polymorphisms, but muscular toxicities seem better related to *SLCO1B1* variant alleles. Their analysis in the general population of patients taking statins could improve treatment adherence and efficacy; however, the cost–efficacy ratio should be carefully evaluated.

## 1. Introduction

Statins are used in clinical practice for hypercholesterolemia treatment and for primary and secondary prevention of cardiovascular diseases and stroke, thanks to their ability to reduce by up 55% plasma low-density lipoprotein cholesterol (LDL-C) [1,2,3]. Statins act principally by competitive inhibition of 3-hydroxy-3-methylglutaryl-coenzyme A reductase (HMGCoAR), thus lowering the synthesis of mevalonate and the production of cholesterol in the liver. The decrease in cholesterol levels within hepatocytes does lead to an augmented synthesis of low density lipoprotein receptors (LDLRs), whose expression on hepatocytes membranes results in a drop in plasma cholesterol and LDL-C [4]. In literature, several works are focused on the potency of statins in the reduction of total cholesterol in patients with hypercholesterolemia [5]; however, statins also reduce the levels of triglycerides [5], up-regulate the endothelial nitric oxide [6,7], and reduce leukocyte–endothelial cell interaction [8,9]. In addition, statins possess pleiotropic effects that help to improve endothelial function, stabilize plaques and decrease oxidative stress and inflammation [10]. Moreover, statins therapy also induces an up-regulation of high-density lipoprotein cholesterol (HDL-C) levels [11].

After the development of the first statin, mevastatin, in the 1970s, the class has enjoyed an exponential market growth. Later, a number of statins have been approved in clinical setting: atorvastatin, cerivastatin, fluvastatin, lovastatin, pitavastatin, pravastatin, rosuvastatin and simvastatin. Several combinations of statins with other agents, such as ezetimibe/simvastatin, are also available and effective [12]. While they share chemical similarities, they are different in terms of pharmacokinetics (absorption, binding to plasma protein, metabolism and solubility), ability to interact with other drugs and ultimately in individual tolerability [13].

Although they are efficacious, in a small percentage of patients the compliance is low, primarily due to the development of side effects [14]. In the literature, several groups have investigated the correlation between statins treatment and adverse drug reactions (ADR). Three meta-analyses on statins used in primary prevention did not show evidence of severe adverse events in patients treated with statins compared to those receiving placebo [15,16,17]. Nevertheless, important adverse reactions have been observed, being the most frequent represented by muscle-related problems [18], increased risk of diabetes mellitus [19] and abnormalities in liver enzymes [20]. In particular, a meta-analysis found a significant correlation between the chronic use of statins and the incidence of diabetes mellitus [19]. Despite these significant findings, the authors highlighted the important role of statins in protecting hyperlipidemic patients from severe cardiac outcomes, further suggesting that statin benefits outweigh those adverse events in high risk cardiovascular patients. In addition, statins therapy might provoke cellular oxidative stress, impairments of mitochondrial function and muscular calcium homeostasis leading to myotoxicity, and consequently to myopathy, myalgia, myositis, and, in rare cases, rhabdomyolysis. Other possible side effects include cognitive loss, neuropathy, pancreatic, hepatic and sexual dysfunctions [14,21].

Some published works suggested a correlation between statin treatment and cancer development [22,23], but this is a controversial issue because other meta-analyses did not find any increase in cancer risk. Indeed, statins appear to have no effect on the risk of lung, kidney, breast, pancreatic, or bladder cancers [24,25,26,27,28], while statins seem to reduce the risk of esophageal, colorectal and gastric cancers, hepatocellular carcinoma, and possibly prostate cancer [29,30,31,32,33].

**Figure 1:**
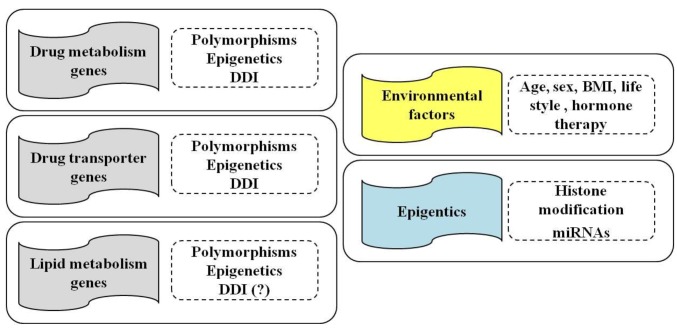
Influence of pharmacogenetics, epigenetics and drug–drug interactions (DDI) on genes involved in drug metabolism, transport and in lipid metabolism (**left** panels) of statins. Environmental and individual factors, such as age, sex and body mass index (BMI), life style (alcohol and smoking) and hormone therapy, as well as epigenetic modifications (histone modifications and miRNAs), also contribute to the efficacy and/or toxicity of statins treatment (**right** panels).

However, one of the major clinical challenges in treating patients with statins is correlated with both their possible side effects and the variable response of patients in terms of efficacy and/or toxicity [1,34,35]. In this regard, genetic factors may contribute to this variability [1,13,36]. Recently, although little evidence does exist regarding epigenetic factors, their involvement in the control of gene expression has emerged as an important mechanism [37] (Figure 1).

## 2. Pharmacogenetics of Statins

Pharmacogenetics is defined as the study of variations in DNA sequence in relation to drug response and adverse events. The understanding of the genetic variations in drug response opens the door to “personalized” or “patient-tailored” medicine through the identification of those patients: (1) who may receive a greater benefit from a pharmacological intervention; and/or (2) are more prone to develop adverse events. Currently, the role of hereditary genetic factors has been recognized as able to influence drug pharmacokinetics and pharmacodynamics. The variability of drug response or the development of side effects followed to statins therapy is highly correlated to the presence of single nucleotide polymorphisms (SNPs). Indeed, the latter ones are able to influence both drug metabolism and transport and may cause inter-individual variability in drug kinetics, efficacy and tolerability. Moreover, variations in genes involved in cholesterol biosynthesis and lipoprotein metabolism have also been identified as causes of inter-individual variation in response to statins.

**Figure 2:**
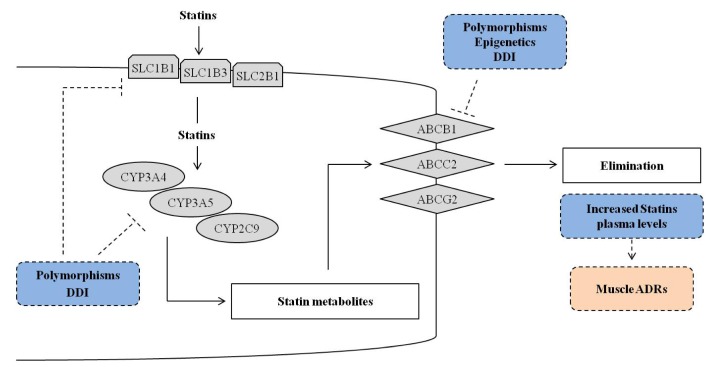
Overview of genetic and epigenetic mechanisms and drug–drug interactions (DDI) that could influence the efficacy of drug metabolism genes (cytochrome, CYP) and drug trans-membrane transporter genes (up-take solute carriers, *SLC* and efflux ATP-binding cassette, *ABC*) that could explain the efficacy and/or toxicity of statins treatment. The dotted line T bar indicates the possible influence of the factors included in the dotted blue rectangle (polymorphisms, epigenetics and DDI) on the cited genes, and the possible effects (dotted pink rectangle).

The most significant and promising SNPs correlated with statins’ toxicity include genes belonging to the family of cytochrome P450, ATPase superfamily, organic anion transporter family, apolipoprotein E (*ApoE*), lipoprotein-associated phospholipase A (LPA) and LDLR (Figure 2 and Table 1, Table 2 and Table 3).

### 2.1. Genes and SNPs Involved in Statin Metabolism

Several drugs, included the HMGCoAR inhibitors, are metabolized by hepatic phase I enzymes, and in particular by cytochrome (CYP) P450 enzymes. Currently, more than 30 different isoforms have been identified; however, the majority of cytochrome-mediated reactions are primarily catalyzed by *CYP2D6* (cytochrome P450, family 2, subfamily D, polypeptide 6), *CYP3A4/5* (cytochrome P450, family 3, subfamily A, polypeptide 4/5), and *CYP2C9* (cytochrome P450, family 2, subfamily C, polypeptide 9) isoforms. In detail, CYP3A4/5 isoenzymes are the major microsomal enzymes that metabolize many statins, including atorvastatin, lovastatin, simvastatin, and to a lesser extent, pravastatin, into active derivates responsible of HMG-CoAR inhibition [13]. CYP2C9 isoenzyme is instead involved in the metabolism of fluvastatin and rosuvastatin, whereas pitavastatin has negligible metabolism through CYP enzymes [106], and it is largely excreted unchanged by the kidneys.

Variations in CYPs alleles can affect the extent of drug metabolism and the activity of CYP450 may vary among individuals. Molecular studies have revealed several *CYP3A4* and *CYP3A5* variants [107]. In particular, inter-individual variability in the expression of *CYP3A* is very high (20- to 40-fold), making the members of this subfamily the major candidates for pharmacogenetic investigations. *CYP3A4* gene is quite strongly conserved, and, although some variants have been described, the in vivo activities of proteins did not show any difference from the wild-type CYP3A4*1. However, the most relevant variant of this gene is CYP3A4*1B that results from an A to G transition in the 5′-flanking region (c.-392A>G, rs2740574). Kajianami et al. have demonstrated that the CYP3A4*1B variant genotype (AG or GG) was significantly associated with higher LDL-C levels in hypercholesterolemic patients treated with 10 mg atorvastatin with respect to subjects carrying the c.-392AA genotype [44], but another study failed to confirm that finding [108]. Furthermore, Becker and colleagues have demonstrated that the CYP3A4*1B was associated with: (1) a lower risk of elevated plasma levels in atorvastatin and simvastatin users compared with the wild-type individuals; and (2) a lower incidence of dose decreases or switching during drug therapy [45].

The variant allele CYP3A5*3 confers low or undetectable *CYP3A5* expression as a result of a single point mutation within intron 3 of the *CYP3A5* gene (c.6986A>G, rs776746) [109]. It has been shown that the metabolism of atorvastatin, lovastatin and simvastatin was significantly lower in individuals carrying at least one CYP3A5*1 allele (expressor) than CYP3A5*3/*3 patients (non-expressor), resulting in decreased liver statin concentrations and, as a consequence, in an impaired hypolipidemic response [48]. On the contrary, another study did not find significant associations among CYP3A4*1B, CYP3A5*3C and the effect of simvastatin on LDL-C levels after two and six months of treatment [49].

More recently, P450 oxidoreductase (POR)*28 polymorphisms have been found related with the lipid lowering response of atorvastatin [110]. Because POR*28 is associated with greater effects on plasma lipids (in particular on total and LDL cholesterol), its polymorphisms may be responsible for the marked inter-individual variability in the lipid-lowering response to atorvastatin [110,111].

Further studies have been addressed to the investigation of the relationship between statin biotransformation and drug-induced adverse effects. Elens and colleagues identified a new *CYP3A4* allele (CYP3A4*22, C>T substitution at position 15389 in intron 6, rs35599367) that was associated with low hepatic *CYP3A4* expression and activity, showing effects on the metabolism of several pharmacological substrates, including statins [112]. However, no associations between CYP3A4*22 and statin effectiveness in reducing the risk for myocardial infarction and on lipid-lowering response in patients with primary hypercholesterolemia have been described [46,47].

Finally, *CYP2C9* is one of the most abundant CYP450 enzymes in the human liver able to metabolize about 15% of clinically used drugs. It is worth noting that the genetic variants CYP2C9*2 and CYP2C9*3 seem to be related with clinical effects of statins. CYP2C9*2 is the missense C>T mutation at position 430, causing an aminoacid substitution at residue 144 (Arg144Cys, rs1799853), whereas the CYP2C9*3 allele is the missense mutation c.1075A>C that leads to Ile359Leu substitution (rs1057910) giving rise to an alteration of the enzyme structure and leading to significantly-reduced enzyme activity. Several studies have shown that the CYP2C9*3 polymorphism has a more pronounced effect than CYP2C9*2. In particular, Mirosevic et al. have shown that patients carrying one or two polymorphic alleles (*2 or *3) had a 2.5 greater odds to develop adverse effects [41]. In relation to statin metabolism by CYP2C9 isoenzyme, Lin et al. have evaluated the associations of *CYP2C9* genetic polymorphisms with the efficacy and safety of rosuvastatin in patients with hyperlipidemia. The results highlighted that patients with the mutant genotype (CYP2C9*1/*3 or *3/*3) showed a higher total- and LDL-cholesterol-lowering effect compared to those with wild-type CYP2C9*1/*1 genotypes [42]. Similar results were also reported in a study by Buzkova et al., who demonstrated an association between CYP2C9*1/*3 genotype and a significant decrease in both LDL-C levels and total cholesterol in comparison with CYP2C9*1/*1 wild-type genotype in hypercholesterolemic patients treated with fluvastatin [43].

### 2.2. Genes and SNPs of Transmembrane Transport

Variations in genes coding for transmembrane transporters are now considered important causes of inter-individual variability of drug kinetics, efficacy, and tolerability (Table 2). Those transmembrane transporters include organic anion and cation transporters, peptide and nucleoside transporters, which are involved in the uptake and excretion of many drugs and their metabolites [37].

Atorvastatin, lovastatin, pitavastatin and simvastatin are substrates of P-glycoprotein, a membrane transporter of adenosine triphosphate (ATP)-binding cassette (ABC) transporter family encoded by the *ABCB1* gene. Further clinical trials demonstrated that atorvastatin, fluvastatin, pravastatin and rosuvastatin are high-affinity substrates for the up-take transmembrane transporters solute carrier organic anion transporter family member 1B1 *SLCO1B1* (Figure 2).

#### 2.2.1. Efflux Transporters

ABC transporters belong to a family of efflux transporters that mediate the translocation of solutes across cellular membranes. These solutes include lipids and sterols, ions and small molecules, drugs and polypeptides. Transporters contain two transmembrane domains responsible for determining substrate specificity, and two nucleotide binding domains able to bind and hydrolyze ATP that supplies energy for substrate translocation. The most common efflux transporters are P-glycoprotein (*ABCB1*, P-gp or multidrug resistance (MDR), encoded by the *ABCB1* gene), breast cancer resistance protein (BCRP or ATP-binding cassette sub-family G member 2, encoded by *ABCG2* gene) and multidrug-resistance-related protein-2 (MRP-2 or ABCC2, encoded by *ABCC2* gene).

#### 2.2.2. ABCB1

ABC transporters, and in particular P-glycoprotein, are widely distributed and expressed in the intestinal epithelium, liver cells, the proximal tubule of the kidney, and the capillary endothelial cells composing the blood–brain barrier and blood-testis barrier. They may influence absorption, excretion, and distribution of drugs. In particular, the most common variants of *ABCB1* gene are represented by c.1236C>T (rs1128503), c.2677G>T/A (rs2032582) and c.3435C>T (rs1045642). Indeed, the *ABCB1* c.1236T allele is associated with a reduction of total cholesterol and LDL-C in patients treated with simvastatin, in comparison with patients with the wild-type c.1236CC genotype. Similar results were observed for the polymorphisms c.2677G>T/A and c.3435C>T, in which the c.2677T/A and c.3435T alleles were observed less frequently in patients suffering from adverse drug reactions (ADRs) than in non-ADRs group [49]. After atorvastatin treatment, *ABCB1* gene showed a reduction of its mRNA expression in particular in the presence of c.2677G>T/A polymorphism. The c.2677T or A allele were related to differences in serum LDL-C and apolipoprotein B (*ApoB*) in response to atorvastatin [50]. The polymorphism c.2677G>T/A was also associated with lipid response in atorvastatin- and pravastatin-treated patients [51]. In another retrospective study, *ABCB1* c.3435CT/TT genotypes were not associated with lipid decrease during simvastatin and atorvastatin therapy [45]. Similar results were obtained more recently by Salaka and colleagues who investigated every possible association between *ABCB1* c.3435C>T polymorphism and changes in triglycerides, LDL-C and HDL-C response in patients taking atorvastatin or simvastatin. Interestingly, changes in HDL-C concentrations but not LDL-C and triglyceride levels were influenced by *ABCB1* c.3435C>T genotype. In particular, patients carrying the *ABCB1* c.3435TT genotype showed a significant decrease in *ABCB1* mRNA and protein levels [53,113,114]. A meta-analysis on hypercholesterolemic patients receiving statins examined the lipid-lowering efficacy and safety associated with the *ABCB1* c.3435C>T. Results indicated that the comparison of CC + CT vs. TT was associated with a significant elevation in the serum HDL-C and total cholesterol levels upon statin treatment, and the *ABCB1* c.3435CC vs. TT variants in homozygotes was correlated with decreases in LDL-C. No significant association was instead observed in serum triglycerides levels [54]. The c.3435C>T polymorphism in *ABCB1* can also explain some heterogeneity of adverse response to statins. The c.3435T allele was more frequent in patients with atorvastatin-induced myalgia [21,55]. In addition, an *ABCB1* c.3435C>T stratification analysis according to treatment duration found an association with a risk of myopathy in patients treated with statins for more than 5 months [54]. Currently, literature does report contrasting data on c.3435C>T SNP, whose association with the effect of statins on cholesterol and lipid levels is still debated. Significant data have been obtained combining the analysis of the three *ABCB1* SNPs (c.1236C>T, c.2677G>T/A and c.3435C>T) in haplotypes. In particular, Becker and colleagues demonstrated that the TTT and CGT haplotypes were associated with total cholesterol and LDL-C levels reduction whilst the wild-type CGC haplotype did not [64]. In a study, Fiegenbaum et al. have shown a reduction of the T-T/A-T haplotype frequency (20%) in patients in whom myalgia developed during simvastatin treatment as compared with those who did not experienced ADR (41.4%), suggesting an association between *ABCB1* gene variants and an increased susceptibility to myalgia [49]. The *ABCB1* haplotype TTT had also a significant influence on inter-subject variability in rosuvastatin pharmacokinetics, with higher values of both maximum plasma concentration (C_max_) and area under the time-concentration curve (AUC) than non-TTT carriers [52]. In addition, the variant allele-carrying genotypes of *ABCB1* c.2677G>T/A and c.3435C>T in hypercholesterolemic patients showed significantly greater LDL-C reduction in response to atorvastatin therapy [57].

#### 2.2.3. ABCG2

*ABCG2* (also known as BCRP) is involved in the transport of a wide range of substrates, included statins [41,61,115,116]. The transporter is expressed in the apical membranes of intestinal epithelial cells, hepatocytes, renal tubule cells and in the endothelial cells that form the blood–brain barrier. The activity of ABCG2 transporter is widely affected by polymorphisms that may play an important role in the pharmacokinetics and toxicity of statins [117]. Indeed, a common SNP, c.421C>A (Gln141Lys, rs2231142), reduces ABCG2 activity and it has been found to be associated with increased systemic exposures to certain statins, including atorvastatin, fluvastatin, simvastatin lactone and rosuvastatin. In particular, the reduced activity of c.421AA ABCG2 in intestinal mucosa may explain the higher AUC values of fluvastatin with respect to individuals carrying the c.421CC genotype [62]. Mirosevic et al. confirmed those data showing that patients with *ABCG2* c.421A variant allele and treated with fluvastatin had 2.75 time greater odds of developing ADR than those carrying the c.421C allele [41]. This polymorphism seems to play an important role in the pharmacokinetic of rosuvastatin [52,63]. Indeed, the effect is strongest for rosuvastatin in subject with c.421AA genotype having 2.0-fold higher statin systemic exposure in comparison with individuals carrying the c.421CC genotype [61]. Similar to rosuvastatin, the exposure to atorvastatin and simvastatin was increased in homozygous carriers of the c.421AA variant [61,64]. In addition, Mirosevic Skvrce et al. have shown that patients with *ABCG2* c.421CA or AA genotypes had 2.9 time greater odds of developing atorvastatin dose-dependent ADRs than c.421CC patients [65]. The mechanism by which *ABCG2* c.421A allele influences statin plasma concentrations is most probably related to an enhanced intestinal absorption and a decreased liver excretion of the drug, hence contributing to an increase in statin bioavailability after oral administration [66]. Although pravastatin has been demonstrated to be a substrate of ABCG2, the c.421C>A polymorphism seems not able to alter the pharmacokinetics of pravastatin [62], suggesting that other ABC transporters may be important for the efflux of pitavastatin and pravastatin. Changes in statins pharmacokinetics could result in a significant impact on drug efficacy and toxicity. Indeed, in patients with hypercholesterolemia treated with rosuvastatin 10 mg/day, the c.421A variant induced a significantly reduction in LDL-C levels compared to individuals carrying the c.421CC [67,68]. Another randomized controlled study in patients with acute coronary syndrome (ACS) found that the *ABCG2* c.421C>A polymorphism had a significant effect on the efficacy and tolerability of rosuvastatin 10 mg. In particular, after a 3-month treatment, patients carrying at least one c.421A allele achieved mean LDL-C levels significantly lower than c.421CC individuals [69]. That relationship was not observed in patients receiving simvastatin 40 mg.

In a larger pharmacogenetic analysis, 125 polymorphisms in 61 candidate genes were analyzed for association with the lipid response to rosuvastatin [67]. Interestingly, rosuvastatin pharmacokinetics was influenced by the presence of the *ABCG2* c.34G>A polymorphism (rs2231137), a non-synonymous SNP located in exon 2 and leading to a valine-to-methionine amino acid change at codon 12 [63]. Furthermore, individuals who were homozygotes or compound heterozygotes for *ABCG2* c.34G>A and c.421C>A had a significant impact on rosuvastatin disposition with a marked alteration of drug pharmacokinetics [63]. However, the c.34G>A polymorphism had no effect on LDL-C response to rosuvastatin within each c.421C>A genotype group, suggesting the association between the c.34G>A SNP and LDL-C response to rosuvastatin is likely to be due to its linkage disequilibrium with the c.421C>A polymorphism [66].

#### 2.2.4. ABCC2

*ABCC2* is expressed in the apical membrane of hepatocytes, proximal tubule of the kidney and enterocytes, where it reduces the gastro-intestinal absorption and facilitates the biliary and renal excretion of its substrates, including several anticancer drugs. Moreover, ABCC2 is involved in the biliary excretion of some statins, as well as pravastatin, pitavastatin and simvastatin. While the expression of *ABCC2* had no correlation with the systemic exposure of simvastatin, higher hepatic expression of *ABCC2* mRNA has been associated with a reduced systemic exposure to the drug [58]. Pravastatin has been identified to be a substrate of ABCC2, and the presence of a rare non-synonymous SNP (c.1446C>G, Thr482Thr) was correlated with changes in pravastatin pharmacokinetics [58]. In addition, other *ABCC2* SNPs could influence *MRP2* expression, including c.-24C>T (rs717620), c.1249G>A (Val417Ile, rs2273697), c.1774T>G, c.3563T>A (Val1188Glu, rs8187694), c.3972C>T and c.4544G>A (Cys1515Tyr, rs8187710), despite none of them seemed significantly associated with alterations in statin pharmacokinetics and drug toxicities [59,60]. On the contrary, Becker and colleagues showed that the c.-24C>T polymorphism and CAC (-24C/1249A/3972C) and TGT (-24T/1249G/3972T) haplotypes were associated with a dose decrease or a switch to another cholesterol-lowering drug in simvastatin users, being these events likely due to adverse effects or a strong reduction in cholesterol levels. For atorvastatin, the researchers did not find any significant relationship among *ABCC2* polymorphisms and the occurrence of ADRs [59]. Finally, the *ABCC2* c.-24C>T SNP significantly influenced pitavastatin pharmacokinetics, and in particular with the reduction of C_max_ and AUC of of the drug [60].

#### 2.2.5. Uptake Transporters

One of the most important up-take transporters is the organic anion polypeptide 1B1 (*OATP1B1*, formerly known as *LST1*, *OATP-C* or *OATP2*) encoded by the *SLCO1B1* gene, and it is involved in the active cellular influx of many endogenous and xenobiotics compounds. *OATP1B1* is expressed predominantly in the basolateral membrane of hepatocytes, where it mediates active intracellular hepatic transport hence influencing the pharmacokinetic and pharmacodynamic profiles of its pharmacological substrates. Indeed, recent works described the role of this transporter in systemic disposition of many drugs [118,119] including statins [120,121]. Currently, a small number of genome-wide association studies (GWAS) have identified loci associated with statin response. Postmus and colleagues performed a meta-analysis of randomized controlled trials and observational studies, identifying *SLCO1B1* variants associated with statin response [74]. Indeed, polymorphisms in *SLCO1B1* gene appear to be important predictors of response to statin, because they are able to induce variations in plasma concentrations of drugs. In particular, two common non-synonymous variants, the c.388A>G and c.521T>C, have been characterized [70]. The *SLCO1B1* polymorphism c.521T>C (SLCO1B1*5, rs4149056) causes the valine-alanine substitution at position 174 (Val174Ala) resulting in a decreased transporter activity, an increased plasma concentration of statins with an attenuation of LDL-C lowering effect. Indeed, that SNP does impact on the pharmacokinetics of simvastatin acid and, to a lesser degree, on parent simvastatin [88], while it may influence the pharmacokinetics of both atorvastatin and rosuvastatin [76]. In addition, several published studies highlighted the association between *SLCO1B1* gene c.521T>C polymorphism and statin-related myophaty risk [77,78]. Further studies have demonstrated that individuals with the c.521CC genotype showed greater systemic exposure to the active simvastatin acid than subjects with the wild-type TT genotype, suggesting that the *SLCO1B1* c.521C allele may enhance the risk of ADRs during simvastatin treatment while decreasing the cholesterol-lowering efficacy of the drug [75]. Wilke et al. reported a marked increase in systemic exposure to simvastatin in homozygous carriers of the c.521C allele, who had a 16.9 times higher risk for myopathy compared with non-carriers [79]. Brunham et al. confirmed the association between the c.521T>C SNP and myopathy in patients receiving simvastatin, whereas no effects were observed in those who were taking atorvastatin [80].

Another common *SLCO1B1* functional variant, the c.388A>G (Asn130Asp substitution, rs2306283), was evaluated in patients treated with statins. The study demonstrated that the SNP was associated with altered effects of these drugs, inducing an increase of LDL-C levels [70] or alterations in other lipid parameters, including HDL or triglycerides [71]. That SNP, also known as SLCO1B1*1b, is associated with higher activity of *OATP1B1* resulting in lower oral bioavailability of statins and in particular of pravastatin [72,73]. On the contrary, a similar trend was not detectable in the pharmacokinetics of rosuvastatin [122]. The *SLCO1B1* c.388A>G and c.521T>C SNPs are in strong linkage disequilibrium [123] and they are associated with possible alterations in the pharmacokinetics of SLCO1B1 substrates. Four common haplotypes have been identified: SLCO1B1*1A (c.388A/c.521T), *1B (c.388G/c.521T), *5 (c.388A/c.521C), and *15 (c.388G/c.521C). In particular, SLCO1B1*5 and *15 haplotypes were mainly associated with increased plasma concentrations of simvastatin acid and, consequently, with a higher risk of muscle toxicity during statin treatment [75]. Recently, Tornio and colleagues showed that SLCO1B1*1B/*1B and *5/*15 or *15/*15 genotypes were significantly related with the systemic exposure to active metabolite lovastatin acid [85]. Interestingly, a GWAS meta-analysis demonstrated that *SLCO1B1* c.1498–1256T>A polymorphism (rs2900478) was in linkage disequilibrium with the c.521T>C SNP [74]. Other variants associated with altered effects of rosuvastatin included *SLCO1B1* c.1498–1331T>C and c.971-901A>G SNPs [70].

Although SLCO1B1 is the primary transporter for the uptake of statins, organic transporter polypeptide 1B3 (*OATP1B3*) and organic anion polypeptide 2B1 (*OATP2B1*) also take part in the process for some specific statins. *OATP2B1* is widely expressed in the heart, kidney, intestine, liver, and placenta where it controls statin uptake. In particular, OATP2B1 is a high-affinity transporter for atorvastatin expressed in the vascular endothelium of the human heart, suggesting its involvement in cardiac uptake of atorvastatin and rosuvastatin [100,101], and the c.1457C>T SNP (Ser486Phe, rs2306168) is one of its common polymorphisms. On the contrary, *SLCO1B3* is exclusively expressed in the liver. Atorvastatin, fluvastatin, pitavastatin and rosuvastatin are SLCO1B3 substrates and genetic variations, including c.344T>G (rs4149117) and c.699G>A (rs7311358), could be potentially involved in statin pharmacokinetics and toxicity [88].

#### 2.2.6. Polymorphisms in Other Genes

A number of genes related to cholesterol biosynthesis and lipid metabolism have been identified as possible causes of variability in response to statins and their toxicities [10], as well as apolipoprotein E (*ApoE*), cholesteryl ester transfers protein (CETP), LDLR and HMGCoAR (Table 3).

*ApoE* and other lipoproteins are responsible for the packaging of cholesterol and other fats, for carrying them through the bloodstream, and for the maintenance of normal cholesterol levels essential for the prevention of disorders that affect heart and blood vessels. *ApoE* gene is located on chromosome 19 and its variants have been studied as risk factors for many different conditions able to influence baseline lipid profiles and statin efficacy. The *ApoE* haplotypes associated with anti-lipidemic, cognitive and thromboembolic phenotypic characteristics are ε2, ε3 and ε4. These haplotypes are produced by *ApoE* gene variants rs7412 (c.472C>T, Cys158Arg) and rs429358 (c.334T>C, Cys112Arg). As summarized in a recent review by Alfonsi et al. [10], several works have described the association among *ApoE*, its variants and statins responses and toxicities. In particular, Thompson and colleagues have examined the association between genes identified as potential modulators of statins and statins response in patients under atorvastatin, fluvastatin, lovastatin, pravastatin or simvastatin therapy [90]. Findings demonstrated that *Apoε2* carriers had a decrease in LDL-C, total cholesterol and triglycerides, whereas HDL-C increased. Since the *Apoε2* protein results in increased hepatic cholesterol synthesis, it may also predispose to stronger inhibition of cholesterol synthesis by statin treatment. In addition, it has been described that *Apoε2* patients had significantly greater LDL-C reduction with atorvastatin and with pravastatin with respect to apoε4 individuals [51]. Moreover, patients with apoε4 allele showed an increased risk to develop atherosclerosis and cardiovascular diseases [93], while experiencing an attenuated LDL-C response to statins in several clinical trials [94,95]. In a meta-analysis aimed at the evaluation of the lipid response to statin treatment among *ApoE* genetic variants (ε2 carriers, ε3 homozygotes and ε4 carriers), significant changes in LDL-C, HDL-C and triglyceride levels were noted for all genotypes, despite those changes did not differ significantly among groups [91]. SNPs in *ApoE* gene have also been associated with progression of coronary disease during statin therapy [124,125]. For example, Gerdes et al. showed that patients carrying the apoE ε4 allele had two-fold higher mortality compared to non-carriers during simvastatin treatment [95].

HMGCoAR is an enzyme involved in the mevalonate pathway, converting the HMGCoAR to mevalonate for the production of cholesterol. Several studies have described the association of the corresponding gene with the therapeutic response to statins [101]. In particular, Chasman et al. analyzed 148 SNPs across 10 genes known to be involved in cholesterol synthesis and statin metabolism, identifying two common polymorphisms, SNP-12 (c.451–174A>T, rs17244841) and SNP-29 (c.2457 + 117T>G, rs17238540) in the *HMGCoAR* gene associated with a decrease in total cholesterol and LDL-C following pravastatin and simvastatin administration [96,97]. More recently, Donnelly et al. confirmed those data showing that individuals heterozygous for the G allele of rs17238540 SNP in the *HMGCoAR* gene may have a suboptimal response to statin therapy (atorvastatin, fluvastatin, pravastatin, rosuvastatin and simvastatin) in terms of total cholesterol and triglyceride lowering [92]. Additionally, others variants of the *HMGCoAR* gene are associated with the variable LDL-C reduction by statin treatment [126]. In a GWAS performed on patients taking pravastatin, carriers of the minor allele (c.2457 + 117G) of *HMGCoAR* SNP-29 had increased cardiovascular events, suggesting a reduced efficacy of the drug [98]. More recently, Swerdlow and colleagues have also demonstrated that the G allele of the *HMGCoAR* c.1368 + 1069G>T (rs17238484) SNP seemed to be associated with an increase in both body weight and risk of type 2 diabetes [99].

Polymorphisms in the *CETP* gene have been associated with both LDL-C response to statin and clinical benefit [100]. CEPT is involved in cholesterol metabolism by transporting: (a) cholesteryl esters back into the liver; and (b) triglycerides from LDL and VLDL to HDL-C. Therefore, the evaluation of *CEPT* genotypes could help to better understand the relevance of this metabolic pathway in association with disease risks. One of the better described polymorphisms is *CEPT* c.118 + 279G>A (also called TaqIB, rs708272), which seems to be associated with both LDL-C response to statins and clinical benefit [1]. In particular, patients with *TaqIB* A allele (*TaqIBB2* variant) showed low CEPT activity, and high HDL-C and ApoA-I concentrations, even if worst cardiovascular outcomes were observed compared with other genotypes. That pattern of response was also observed with the c.1264G>A and c.-629C>A polymorphisms [127]. In statin-treated patients affected by cardiovascular diseases, the genetic variation conferring low CEPT levels was associated with increased mortality, suggesting that the efficacy of statin therapy could depend on *CEPT* genotype and *CEPT* expression [128].

The *LDLR* gene was studied to evaluate every possible correlation among statins and adverse drug reactions [101]. This transporter mediates endocytosis of lipoprotein, in particular LDL-C, and apoE protein. Two SNPs within the *LDLR* gene, the c.44857C>T (rs1433099) and c.2052T>C (rs5925), were found to be associated with lipid-lowering response. The first polymorphism has been described by Polisecki and colleagues, who noted a significant association between lower levels of LDL-C and cardiovascular disease in pravastatin-treated patients with coronary heart disease [102]. The latter SNP, the *LDLR* c.2052T>C, seems capable to significantly influence the LDL-C response to pravastatin in patients with hypercholesterolemia [103].

At least, kinesin-like protein 6 (KIF6) is involved in intracellular transport of organelles and several molecules, as well as protein complexes and mRNAs. Furthermore, some results suggest a role in statin response. Indeed, literature reports that the polymorphism rs20455 (Trp719Arg substitution) in the *KIF6* gene is strongly associated with coronary heart disease, and that carriers of 719Arg allele receive significantly greater benefit from intensive statin therapy than non-carriers [98,104,105].

## 3. Statins and Epigenetics

Epigenetics represents the study of heritable and reversible changes in DNA able to influence gene expression and chromatin structure without entail a change in DNA sequence [129]. The epigenetic modifications described in literature include DNA methylation, histone modifications, and non-coding RNAs (ncRNAs) [37] (Figure 1 and Figure 2).

As described above, statins exert many pleiotropic effects, including beneficial effects on endothelial function and blood flow, decreased LDL-C oxidation, enhanced atherosclerotic plaque stability, decreased vascular cells proliferation and platelet aggregation, reduced vascular inflammation. It is worth noting that epigenetic mechanisms could contribute in the explanation of those pleiotropic effects. Indeed, simvastatin and fluvastatin might control atherosclerotic inflammation by affecting histone modifications, through reducing the acetylation of histone H3 and H4 as well as phosphorylation of histone H3, and partly restoring global histone deacetylase (HDAC) activity to prevent the loss of binding of HDAC-1 and -2 at the promoter region of inflammatory genes [129,130].

Nevertheless, several GWASs indicate that only a fraction of cardiovascular diseases is associated with genetic variations localized in protein-coding genes, whereas the majority are located in genomic regions that could express non-coding RNAs, including micro RNAs (miRNAs). miRNAs play an important role in the modulation of gene expression in several biological and cellular processes by down-regulating the translation of target mRNAs [131]. Many miRNAs are implicated in cardiovascular diseases and in the development of atherosclerosis. miR-33 is widely expressed in different cell types and tissues and it has been reported to contribute to the regulation of cholesterol homeostasis through the modulation of genes involved in cellular cholesterol transport and cell efflux, such as *ABCA1* and apolipoprotein A1, respectively. At the same time, miR-33 is capable to target *ABCG1* and to reduce cholesterol efflux to HDL [132,133,134]. In addition, Allen and colleagues demonstrated that miR-33 is transcriptionally induced following treatment with statins, assuming that this miRNA might account for some of the side effects of these drugs, as well as hepatotoxicity. Indeed, statin-induced miR-33 can repress both *ABCB11* and *ATP8B1* that lead to a decrease in bile secretion, and it could play a pivotal role in hepatic response to statins by coordinating the expression of several sterol transporters [135]. Finally, the disruption of miR-33 pathways could prevent statin-induced hepatotoxicity.

As previously reported, statins may protect the cardiovascular system by improving the endothelial function, increasing nitric oxide (NO) levels and mRNA expression of the endothelial nitric oxide synthase (*eNOS*). Recent evidence also describes the modulation of NO release by miRNAs. Cerda and colleagues showed that statins, in particular atorvastatin and simvastatin, increased NO levels and eNOS mRNA expression. In addition, the authors also provided new evidence about the role of miR-221, -222 and -1303 on NO release mediated by statins [136]. As previously described, atorvastatin reduced the expression of miR-221 and miR-222 in patients with coronary artery disease [137], whereas simvastatin down-regulated miR-221 only. Interestingly, they showed that ezetimibe, an inhibitor of cholesterol absorption, reduced miR-221 expression that could be dependent on the regulation of intracellular cholesterol [136]. miR146a/b resulted also implicated in the pathogenesis and clinical manifestations of atherosclerosis, while it seems modulated by combined treatment with statins, in particular with atorvastatin, in patients at high risk of coronary heart disease [138]. Atorvastatin also control *ABCB1* expression via miRNAs. Rodrigues et al. found that miR-491-3p targets 3′-UTR of *ABCB1* and that its expression was up-regulated after atorvastatin exposure, suggesting that this miRNA may be a potential target of atorvastatin to control *ABCB1* expression [139]. More recently, it has demonstrated that statins may inhibit aberrant miR-133a expression in the vascular endothelium to prevent endothelial dysfunction and consequent cardiovascular diseases by targeting GTP cyclohydrolase 1 (GCH1) [140]. Finally, the incidence of cardiovascular events and levels of inflammatory markers in patients with acute coronary syndromes receiving percutaneous coronary intervention may be decreased by a pre-treatment with high doses of rosuvastatin [141]. The suppression of miR-155/SHIP-1 signaling pathway may explain, at least in part, that finding. Another miRNA, miR-21, plays a major role in the regulation of the anti-inflammatory effects of lovastatin at the cellular level [142].

## 4. Statins, Individual and Environmental Factors

The risk and severity of adverse drug reactions of statins and their therapeutic benefit are also related to non-genetic factors, including individual and environmental factors (e.g., age, sex, race/ethnicity, body mass index and obesity, diet, physical activity, sedentary time, and air pollution) and concomitant medications (see paragraph below) (Figure 1).

Miltiadous et al. have studied the effect of individual factors such as age, sex, smoking habit and body mass index (BMI) on the response to statin therapy and, of consequence, on their toxicity in patients with familial hypercholesterolemia treated with atorvastatin 20 mg/day. The results showed that none of these environmental factors affected the lipid-lowering response to statin therapy [143]. Other studies reported that smokers have smaller statin-induced LDL-C decrease compared with nonsmokers, with a consequent increased risk of ischemic heart diseases [144].

Dietary factors may also contribute to the action of statins and their adverse reactions. Proteins, low- or high-content lipid diets, carbohydrates, and in particular fibers, vitamins D and PP, and alcohol consumption could influence the efficacy and the tolerability of statins [145].

It also seems that inflammation might cause statin resistance [146]. Indeed, Robertson and colleagues described that, despite lower rates of statin use, the patients with active rheumatoid arthritis (RA) displayed a greater mean reduction in total cholesterol and LDL-C during the five years preceding diagnosis when compared with the control population [147]. However, changes in lipid plasma concentrations in RA patients seems to reflect an inverse correlation with the severity of inflammation (the “lipid paradox”) while anti-inflammatory drugs may increase LDL-C, total cholesterol and alter the composition of lipoprotein particles (a pharmacodynamic DDI). Those factors may explain the complexity and perhaps the difficulty to evaluate statin effects in RA population, both in brief and long term follow up (i.e., reduction of LDL-C and the risk of cardiovascular disease, respectively). Indeed, anti-inflammatory therapies increase lipid levels in these patients but without leading to a rise in the number of cardiovascular events [148].

## 5. Clinically Relevant Drug–Drug Interaction with Statins

Subjects undergoing a statin treatment often need co-administration with other drugs to improve their general health condition. Drug–drug interactions (DDIs) have to be taken into consideration as potential cause of muscle-related toxicity in patients administered with statins (Figure 1 and Figure 2). In particular, the inhibition of CYP isoenzymes and transporters is an important cause of drug interaction. Competitive inhibitions between drugs are common and could alter the disposition of statins, leading to increased plasma levels and a higher risk of adverse events [149].

With regards for the risk of DDIs, the fold increase in the statin AUC has been used as classifier of the level of DDIs and defined as minor (>1.25–<2.0), moderate (≥2–4.9), or severe (≥5) [150].

### 5.1. Statins and Cardiovascular-Anti-Platelet/Anti-Coagulant Drugs

Patients who received co-prescription of calcium channel blockers, known CYP3A4 inhibitors (i.e., amlodipine, diltiazem, felodipine, nicardipine, nifedipine, and verapamil), and CYP3A4-metabolized statins (i.e., lovastatin, simvastatin, and atorvastatin) had significantly higher risk of acute kidney injury, hyperkalemia, acute myocardial infarction, and acute ischemic stroke than those who received non-CYP3A4-metabolized statins (i.e., fluvastatin, rosuvastatin, and pitavastatin) [150].

The same principle can be applied to ticagrelor, an antiplatelet agent metabolized by CYP3A4 and a substrate of ABCB1. Ticagrelor produces a slight increase in the atorvastatin C_max_ and AUC (23% and 36%, respectively), whereas it causes a more pronounced increase in simvastatin C_max_ and AUC (81% and 56%, respectively) [151]. In line with this evidence, combination with ticagrelor is acceptable for atorvastatin without dose limitation, whereas maximum daily dose of simvastatin should be 40 mg [151].

Timing of drug-related adverse events is of paramount importance in predicting the clinical significance of a DDI. This is also true for statins because serious adverse effects of this drug class generally occur several days after drug administration. For example, even though clopidogrel has the potential to interact with statins by inhibiting OATP1B1 and CYP3A4, the clopidogrel dosing protocol of 300 mg followed by 75 mg daily has proved to induce a transient effect (at the loading phase) on the exposure to simvastatin in healthy volunteers [152]. Since the clinical relevance of clopidogrel-simvastatin interaction at maintenance doses of clopidogrel appears to be more important than that at the loading dose, the use of therapeutic doses of clopidogrel should not affect the total exposure to simvastatin to a clinically relevant extent.

The novel oral anticoagulant rivaroxaban has also the potential to interact with statins because it is metabolized via CYP3A4 and is a substrate for ABCB1 [153]. However, 20 mg rivaroxaban had no effect on the steady-state atorvastatin pharmacokinetics [154]. Warfarin is commonly prescribed to patients in treatment with statins in order to prevent atrial fibrillation or deep vein thrombosis. In a retrospective cohort study on 1.686 patients who received concomitantly statin and warfarin, the incidence of gastrointestinal bleeding and the reduction in mean cholesterol levels followed the order rosuvastatin > atorvastatin > simvastatin > pravastatin. Findings of this study (although with limitations) suggest that a strong reduction in cholesterol levels (particularly for high-potency statins) might weaken cell membrane in gastrointestinal mucosal cells thus favoring the occurrence of gastrointestinal bleeding [155].

### 5.2. Statins and Immunosuppressant Drugs

The immunosuppressant drugs cyclosporine and tacrolimus are extensively metabolized by hepatic and intestinal CYP3A4 and act as both inhibitors and substrates of ABCB1 [156]. Cyclosporine also inhibits the hepatic uptake transporter OATP1B1 [157]. Inhibition of CYP3A4 and/or transport proteins in the gastrointestinal tract and/or in the hepatic tissue results in enhanced statin bioavailability. In heart transplant recipients under immunosuppressant drugs, statins are commonly used to reduce rejection associated with progressive hemodynamic worsening. This drug combination produces 6- to 15-, 6- to 8-, and 5- to 20-fold increase in AUC of atorvastatin, simvastatin, and lovastatin, respectively. In contrast, fluvastatin is mainly metabolized by CYP2C9, a condition that reduces the risk of statin-related adverse events in patients taking cyclosporine [158]. Noteworthy, while inhibition of first-pass effect of lovastatin or simvastatin increased oral availability with high risk of muscle-related toxicity, such an effect may be buffered for atorvastatin because both parent drug and metabolites are active [159]. According to this principle, combination of immunosuppressant drugs should be avoided with simvastatin and lovastatin, whereas it may be considered with atorvastatin by limiting daily dose [160]. Finally, it should be kept in mind that cyclosporine may also increases exposure to non CYP-metabolized statins (e.g., pravastatin, pitavastatin and rosuvastatin) via inhibition of OATP1B1 [13].

### 5.3. Statins and Anti-Microbial/Anti-Viral Drugs

Macrolide antibiotics, including erythromycin and clarithromycin, are potent CYP3A4 inhibitors at clinically relevant doses. These drugs have been shown to interact with statin metabolism by increasing the AUC of the victim drugs by about 1.5- to 12-fold [158]. Co-administration of azole anti-fungals with statins has also been reported to significantly increase the risk of statin-related rhabdomyolysis in healthy individuals and patients [161]. The mechanism responsible for this effect relies on a strong CYP3A4 inhibition, as demonstrated for itraconazole and ketoconazole that increase the AUC of lovastatin and simvastatin by up to about 20-fold [158].

Clinically relevant DDIs have been reported for statins combined with oral direct-acting antiviral agents that target various steps in the HCV life cycle. Co-administration of pravastatin with the 2D regimen of ombitasvir (25 mg) and paritaprevir-ritonavir (150/100 mg) increased the pravastatin C_max_ and AUC mean values by 43% and 76%, respectively. C_max_ and AUC of rosuvastatin also increased in the presence of the 2D regimen by 161% and 33%, respectively [162]. As far as the potential mechanism of DDI is concerned, paritaprevir and ritonavir are primarily metabolized by CYP3A, whereas ombitasvir is metabolized by amide hydrolysis followed by oxidative metabolism. Furthermore, paritaprevir is a OATP1B1/B3 inhibitor, and paritaprevir and ritonavir are potential inhibitors of ABCB1 and ABCG2 [162]. This appears particularly relevant from the clinical point of view because OATP and ABCG2 have been reported to be involved in statin uptake and metabolism [163]. HIV positive patients treated with protease inhibitors (and CYP3A4 inhibitors), such as atazanavir, can need statins to reduce the high risk for dyslipidemia, insulin resistance and cardiovascular disease [164]. Pitavastatin-atazanavir combination has been demonstrated to be safe in HIV-infected patients with no hepatotoxicity signs or significant increase in creatine phosphokinase levels [165].

### 5.4. Other Interactions

It is not clear whether DDIs between oral anti-diabetic agents and statins may have clinical relevance in terms of efficacy or safety. In an open-label study carried out on twenty healthy subjects, simvastatin 40 mg/day was administered alone for six days, followed by co-administration with the dipeptidyl peptidase-4 inhibitor linagliptin 10 mg/day for six days. Simvastatin AUC and C_max_ following co-administration of linagliptin were 134.2% and 110% higher than those obtained in the simvastatin alone group; these changes were not considered to be clinically relevant [166].

Gemfibrozil, a lipid-lowering drug belonging to fibrates, is a CYP2C8-inhibitor that has been associated to a remarkable increase in the AUC of cerivastatin with clinical consequences leading to its withdrawn from the market in 2001 [158].

## 6. Conclusions

Statins are generally effective and well tolerated by the majority of patients. Nevertheless, there is a significant minority of individuals showing a reduced benefit and an increased susceptibility to develop severe side effects, the latter being a possible cause of a long-term poor compliance. Notably, statin-associated myopathy represents a growing problem given the increasing use of statins in worldwide population.

This review focused on the pharmacogenetics of statins, including cytochrome P450 enzymes (CYP2C9, CYP3A4 and CYP3A5), uptake (SLCO1B1) and efflux transporters (ABCB1, ABCG2 and ABCC2), showing how changes in nucleotide sequences may influence pharmacokinetics, efficacy and tolerability of these drugs. However, the risk of severe ADR from statins may be increased by other causes, as well as individual and environmental factors that should be taken into consideration when planning clinical trials and analyzing their findings. For example, DDI and/or genetic polymorphisms have been shown to increase statin exposure causing a high risk of developing myopathy.

Most classical DDI involve the alteration of both drug biotransformation (especially CYP450 isoenzymes) and transmembrane transporters. Indeed, the pharmacogenetic of statins identifies cytochromes as one of the possible causes of the variable tolerability, but this does not represent the easiest target for investigation. Indeed, several CYP isoforms with their multiple allelic variants make difficult to predict the relationship between prescription of statins and risk of toxicities. On the contrary, there is very strong evidence that transmembrane transporters can have a major role in determining the association between statins and myopathy. In particular, pharmacogenetic studies have shown that ABC and SLC transporters are critically involved in the absorption and disposition of statins and that inter-individual differences in drug response and toxicities have solid pharmacogenetic bases. The presence of polymorphic variants in genes codifying for efflux and uptake transporters is related to the development of side effects due to altered statin passage through cell membranes. Efflux transporters, such as ABCB1, ABCC2 and ABCG2, have been described to significantly influence the systemic pharmacokinetics of statins, while some of their correlated genetic variants are capable to influence the efficacy and the tolerability of those drugs. At the same time, uptake transporters have similar effects. Indeed, the data discussed in this review seem to suggest that muscular toxicities are better related to *SLCO1B1* variant alleles (SLCO1B1*5, c.521C), able to reduce the hepatic uptake of statin, to increase the systemic exposure and, consequently, to augment muscular side effects.

The pharmacogenetic analysis in the general populations of patients taking statins could improve treatment adherence and efficacy. As a result of those analyses, every patient could receive his/her own genetic signature associated with individual pharmacokinetics of statins and the corresponding absolute risk for toxicities. However, the analyses could also result in the relative risk of side effects, when coadministered drugs may significantly influence statin disposition and could worsen that risk. Nevertheless, there are several limitations to this approach, as well as the fact that the presence or absence of the polymorphic variant for a specific locus (a dycothomic variable) may not fully explain the association between genes and tolerability, leaving a non-negligible uncertainty area. Possible solutions could be represented by the analysis of pharmacogenetic (and epigenetic) factors along with other variables, such as age, co-morbidities, potential DDI, liver and kidney functions, smoking, etc. Appropriate statistical methods and tools are available to further explore the pharmacogenetics of statins in that direction.

In common with other pharmacological-pharmacogenetic studies, the identified predictive markers have to be confirmed in large prospective interventional clinical studies. This represents a formidable hurdle because the occurrence of severe toxicities has a low frequency, meaning that large population of patients should be enrolled to observe a significant reduction of toxic events (or just changes in laboratory biochemical indexes, which are more feasible surrogate endpoints). On the other way, statin efficacy may be based on both early and late clinical endpoints, as well as the improvement in lipid profile and the reduction of cardiovascular events, respectively. The latter need longest follow up periods, making the validation of predictive markers more cumbersome. However, meta-analyses and the involvement of general practitioners could help in overcoming that issue.

Third, in the last years, more attention is paid to the real world evidence of interventions to improve healthcare, as well as drugs, devices and biomarkers. Therefore, the transfer of those predictive factors in real world practice should result in certain advantages for patients, caregivers and other stakeholders, but the achievement of those improvements could be influenced by the availability of analytical platforms and personnel expertise.

In conclusion, pharmacogenetic analyses represent an optimal opportunity to investigate and discover markers that may predict the efficacy and the tolerability of statins. However, their pleiotropic effects and the reciprocal influence of several biochemical pathways, especially in comorbid patients, significantly support a wider and careful strategy during trial planning and conduction. Furthermore, poor knowledge of confounding factors could frustrate every effort. Finally, as stated above, confirmative prospective studies and real world evidence represent additional requirements before considering every patient stratification approach suitable for routine application to anticipate drug efficacy and tolerability of statins.

## Figures and Tables

**Table 1:** Drug metabolism genes that influence the efficacy and/or toxicity of statins.

Gene	Location	Polymorphism	ADRs	Efficacy	References
*CYP2D6*	22q13.2	CYP2D6*4	√	√	[38,39]
		c.1846G>A-rs3892097	[A, S]	[A, F, P, R, S]	
		CYP2D6*10	–	√	[40]
		c.188C>T-rs1065852		[S]	
*CYP2C9*	10q23.33	CYP2C9*2	√	√	[41]
		c.430C>T-rs1799853	[F]	[F]	
		CYP2C9*3	√	√	[41,42,43]
		c.1075A>C-rs1057910	[F, R]	[F, R]	
*CYP3A4*	7q21.1	CYP3A4*1B	√	√	[44,45]
		c.-392A>G-rs2740574	[A, S]	[A, S]	
		CYP3A4*22	–	√	[46,47]
		c.522-191C>T-rs35599367		[A, F, P, R, S]	
*CYP3A5*	7q21.1	CYP3A5*3	–	√	[48]
		c.6986A>G-rs776746		[A, L, S]	

A: atorvastatin; F: fluvastatin; L: lovastatin; P: pravastatin; R: rosuvastatin; S: simvastatin; ADRs: adverse drug reactions.

**Table 2:** Drug transporter genes that influence the efficacy and/or toxicity of statins.

Gene	Location	Polymorphism	ADRs	Efficacy	References
*ABCB1*/*MDR-1*	7q21.12	c.1236C>T-rs1128503	√	√	[49]
			[S]	[S]	
		c.2677G>T/A-rs2032582	√	√	[49,50,51,52]
			[S]	[A, P, S]	
		c.3435C>T-rs1045642	√	√	[21,45,49,53,54,55]
			[A, S]	[A, S]	
		Haplotype TTT	√	√	[49,52,56,57]
			[S]	[A, R, S]	
*ABCC2*/*MPR-2*	10q24.2	c.1446C>G	–	√	[58]
				[P]	
		c.-24C>T-rs717620	–	√	[59,60]
				[Pi, S]	
*ABCG2*/*BCRP*	4q22.1	c.421C>A-rs2231142	√	√	[41,52,61,62,63,64,65,66,67,68,69]
			[A, F, R]	[A, F, R, S]	
		c.34G>A-rs2231137	–	√	[63,66]
				[R]	
*SLCO1B1*/*OATP1B1*	12p12.1	SLCO1B1*1B	–	√	[70,71,72,73]
		c.388A>G-rs2306283		[P]	
		SLCO1B1*5	√	√	[70,74,75,76,77,78,79,80,81,82,83]
		c.521T>C-rs4149056	[C, S]	[A, P, R, S]	
		SLCO1B1*15	√	√	[75,84,85,86,87]
		(c.388G-c.521C)	[L, S]	[L, P, Pi, S]	
*SLCO1B3*/*OATP1B3*	12p12.2	c.344T>G-rs4149117	–	√	[88]
				[A, F, Pi, R]	
		c.699G>A-rs7311358	–	√	[88]
				[A, F, Pi, R]	
*SLCO2B1*/*OATP2B1*	11q13.4	c.1457C>T-rs2306168	–	√	[81,88,89]
				[A, R]	

A: atorvastatin; F: fluvastatin; L: lovastatin; P: pravastatin; Pi: pitavastatin; R: rosuvastatin; S: simvastatin; ADRs: adverse drug reactions.

**Table 3:** Other genes that influence the efficacy and/or toxicity of statins.

Gene	Location	Polymorphism	ADRs	Efficacy	References
*APOE*	19q13.32	ApoE ε2	–	√	[51,90,91,92]
		(c.334T-c.472T)		[A, F, L, P, S]	
		ApoE ε4	√	√	[91,93,94,95]
		(c.334C-c.472C)	[P, S]	[P, S]	
*HMGCoAR*	5q13.3	SNP12	–	√	[96,97]
		c.451-174A>T-rs17244841		[P, S]	
		SNP29	√	√	[92,96,97,98]
		c.2457 + 117T>G-rs17238540	[P]	[A, F, P, R, S]	
		c.1368 + 1069G>T-rs17238484	√	–	[99]
*CEPT*	16q13	TaqIB	√	√	[1,100]
		c.118 + 279G>A-rs708272	[P]	[P]	
*LDLR*	19p13.2	c.44857C>T-rs1433099	√	√	[101,102]
			[P]	[P]	
		c.2052T>C-rs5925	√	√	[103]
			[P]	[P]	
*KIF6*	6p21.2	c.2155T>C-rs20455	√	–	[98,104,105]
			[P]		

A: atorvastatin; F: fluvastatin; L: lovastatin; P: pravastatin; R: rosuvastatin; S: simvastatin; ADRs: adverse drug reactions.
